# Cellular therapy in older adults with relapsed/refractory diffuse large B-cell lymphoma

**DOI:** 10.3389/fonc.2024.1481950

**Published:** 2024-10-22

**Authors:** Naseem Esteghamat, Aaron Tsumura, Gabriel Marquez-Arreguin, Joseph Tuscano

**Affiliations:** ^1^ Division of Malignant Hematology, Cellular Therapy and Transplantation, University of California Davis Comprehensive Cancer Center, Sacramento, CA, United States; ^2^ Division of Hematology & Oncology, University of California Davis Comprehensive Cancer Center, Sacramento, CA, United States; ^3^ Schoool of Medicine, University of California, Davis, Sacramento, CA, United States

**Keywords:** CAR T, CAR T cell, elderly, DLBCL - diffuse large B cell lymphoma, autologous transplant (ASCT), bispecific antibody (bsAb)

## Abstract

Relapsed/Refractory (R/R) diffuse large B-cell lymphoma (DLBCL) is an aggressive disease with poor prognosis and limited therapeutic options. High-dose chemotherapy with autologous hematopoietic stem cell transplantation (autoHCT) was historically the curative-intent treatment for patients who demonstrated chemosensitivity to salvage therapy. However, a significant portion of patients do not make it autoHCT due to disease progression or overall fitness and eligibility. This is of particular concern in the older adult population. In recent years, significant advances in cellular therapies including chimeric antigen receptor (CAR) T-cells and bispecific antibodies, in addition to improvement in autoHCT tolerability, have allowed for additional treatment options for patients with R/R DLBCL. These novel therapies offer the potential for durable remissions and cure, and should be considered in older patients. We present a review focused on the safety and efficacy of cellular therapies in the older adult population with R/R DLBCL.

## Introduction

DLBCL is the most common subtype of non-Hodgkin lymphoma (NHL), representing about 30% of cases. It is curable with multiagent chemo-immunotherapy in approximately 60% of patients depending on disease characteristics including molecular classification, genetic rearrangements, stage, age-adjusted international prognositic index (IPI), and age at diagnosis (1). Patients who have disease refractory to first line therapy or relapse after initial treatment have poor prognosis, and those with primary refractory disease have particularly poor outcomes, with estimated overall survival of approximately 6 months (2). Historically, patients with R/R disease deemed eligible are treated with salvage chemotherapy, and if they demonstrate chemosensitive disease, go on to receive high-dose chemotherapy with autoHCT. However, elderly patients are often not eligible candidates for autoHCT, and approximately 40-50% of patients are estimated to eventually relapse after autoHCT, leaving limited treatment options post-transplant, particularly in older, frail patients (2).

Recent advances in cellular therapies have changed the treatment landscape in R/R DLBCL. These novel therapies include CAR T-cells and bispecific antibodies. High-dose chemotherapy and autoHCT has also historically been limited to younger patients, although risk stratification and identification of factors associated with morbidity and mortality have also extended this as a treatment option to an older adult population. These cellular therapies offer promising response rates and potentially durable remissions with different toxicities compared to traditional chemotherapy; thus, they should be considered for older adults who are deemed appropriate candidates for these treatments.

The median age at diagnosis of DLBCL is 67 years (3); however, older patients are often underrepresented in cancer clinical trials despite accounting for a larger population of the prevalence of these diseases (4, 5). Thus, the ability to apply these data to the older adult population represents a clinical challenge. With the many recent practice-changing advances in cellular therapies for R/R DLBCL, the older adult patient population should still be considered for these potentially curative therapies, with attention paid to toxicity, survival, and non-relapse mortality. We present a review of the literature for cellular therapies in the older adult population with R/R DLBCL.

## Chimeric antigen receptor T-cells

CAR T-cell therapy has become a well-established treatment approach for R/R DLBCL within the last decade, with FDA approvals for axicabtagene ciloleucel (axi-cel), tisagenlecleucel (tisa-cel), and lisocabtagene maraleucel (liso-cel). These products were approved based on their pivotal trials ZUMA-1, JULIET, and TRANSCEND NHL-001, respectively. While all three CAR T-cell products are CD19-directed, their contructs differ. Axi-cel comprises an anti-CD19 single chain variable fragment (scFv) linked to a CD28 and CD3-zeta co-stimulatory domain, while both tisa-cel and liso-cel are comprised of an anti-CD19 scFv linked to a 4-1BB (CD137) and a CD3-zeta co-stimulatory domain. Axi-cel and liso-cel have FDA approval in patients with DLBCL who relapse within 12 months of first-line therapy, while tisa-cel is FDA approved for DLBCL patients who have relapsed after two or more lines of systemic therapy.

As we have gained more experience and optimization of this specialized cellular therapy, rates of the highly morbid side effects of Cytokine Release Syndrome (CRS) and Immune Effector Cell-Associated Neurotoxicity Syndrome (ICANS) have been reduced. Early recognition and effective management of these syndromes have made CAR T-cells both effective and safe, even in patients with advanced age, poor fitness, or comorbid conditions. Importantly, in all three aforementioned landmark studies, though not statistically significant, subgroup analyses show numerically better objective response rates (ORR) and complete responses (CR) in patients >65 compared to their younger counterparts. ZUMA-1 (axi-cel) reported an ORR of 92% vs. 79%, CR 75% vs 53%, and mPFS 13.2 mo vs 5.6 mo (6). JULIET (tisa-cel) reported an ORR of 59% vs 49% (7). TRANSCEND NHL-001 (liso-cel) reported an ORR of 75.9% vs 70.3% as well as CR of 60.2% vs 48.0% (8). These data suggest that elderly patients (≥65) gain to have more benefit from CAR T than their younger counterparts. Rates of grade >3 CRS were 13%, 22%, and 1%, while rates of grade >3 ICANS were 31%, 12%, and 13%, respectively (6–8). Management for CRS and ICANS among the three agents is largely similar with one exception to consider IV Dexamethasone for early onset grade 1 CRS (<72 hours) with liso-cel based on NCCN guidelines (9).

Following these observations, the PILOT study was a phase 2 trial assessing Liso-cel as second-line therapy for patients with R/R DLBCL who were not considered for Hematopoietic Stem Cell Transplantation (HSCT). Though they were open to enroll patients 18 years and older, 79% of patients were 70 years or older (33% 70-74, 46% ≥75). With a median age of 74 years, they reported an ORR of 80% and CR of 54%. Subgroup analysis, although not statistically significant, showed greater ORR and OS when the groups were compared at cutoffs of 65 years, 70 years, and 75 years (i.e <65 vs >65, <70 vs >70, and <75 vs >75). There were no treatment related deaths and rates of CRS and ICANs were similar to prior studies (10). Additionally, a planned subgroup analysis of the ZUMA-7 trial compared Axi-cel to standard-of-care (SOC) chemoimmunotherapy as second-line treatment for patients aged >65 with DLBCL who relapsed within 12 months after first-line therapy. The analysis reported an ORR of 88% with CAR T vs 52% SOC chemoimmunotherapy. The CR rate was 75% vs 33%. Though grade >3 AEs were higher in the CAR T arm (94% vs 82%), quality of life scores at day 100 and day 150 were also higher, thus leading to the FDA approval of axi-cel as second-line for patients with DLBCL who relapse within 12 months after first line therapy (11). Lastly, Johnson et al., performed a retrospective analysis evaluating clinical outcomes and treatment toxicity in older patients who received CAR T at Massachusetts General Hospital. In comparing patients aged >75 vs. 65-74, they reported no difference in rates of CRS or ICANS, as well as hospital readmissions or Intensive Care Unit (ICU) admissions within 30 days of their CAR T-cell infusion. Median hospital length of stay was similar and there was no associated OS or event-free survival (EFS) benefit when controlling for CAR T-cell product, performance status, age ≥ 75, as well as other variables (12). Overall, these findings demonstrated safety and efficacy of CAR T-cells over SOC chemoimmunotherapy for this older age population.

Despite the approval of CAR T-cell therapies, Chihara et al. found a significant under usage of CAR T-cells in elderly patients, especially in patients aged >75. After querying the Medicare fee-for-service claims database from October 2015 thru December 31, 2020, they reported the low utilization rates of CAR T-cells in elderly patients by age; 19% in patients 65-69, 22% in patients 70-74, and 13% in patients 75 or older. This was despite their findings of median EFS of 43%, 52%, and 34% for patients 65-69, 70-74, and >75, respectively (13). They found a median OS of 17.1 months with no significant differences based on age groups. To our knowledge, there has not been a similar assessment of utilization rates of CAR T-cells in DLBCL patients younger than 65.

It is possible that some of these patients were taken to autoHCT, in alignment with Shadman, et al. reporting improved progression free survival (PFS) and overall survival (OS) in chemotherapy-sensitive relapsed DLBCL treated with autoHCT vs CAR T (14). However, notably in their retrospective study, there were no reported subgroup analyses based on age groups. Akhtar, et al. performed a similar retrospective propensity-matched analysis concluding that CAR T-cell therapy has similar efficacy to autoHCT, specifically in older patients aged >65 years. All patients had chemosensitive R/R DLBCL with at least a partial response (PR) to salvage therapy prior to cellular therapy. They found no difference in 1-year PFS (52% for autoHCT vs 51% for CAR T) and no difference in 1-year OS (71% vs 73%). Similar results were seen when the age cutoff was increased to >70 years and <70 years. Notably, they did find that for patients age <65, CAR T-cells had inferior PFS and OS as compared to autoHCT with hazard ratio (HR) of 1.79 (p= 0.02) and 2.3 (p=0.004), respectively (15). Recently, Guffroy et al., reported on their retrospective analysis of real-world outcomes of anti-CD19 CAR T-cell therapy in patients aged >75 with R/R large B-cell lymphoma registered in the French DESCAR-T registry. Their findings were presented at the European Hematology Association 2024 (EHA2024). They retrospectively compared patients who were either <75 years old or >75 (n=1399 and n=125, respectively) and found similar rates of ORR and CR, as well as similar PFS and OS. The most significant difference was a higher rate of non-relapse mortality (NRM) in patients >75 compared to those <75 (19.5% vs 8.1%), but lower rates of lymphoma-related mortality. This difference was mainly seen in late NRM (>28 days post-infusion), but did not translate into a lower OS rate. The main cause of NRM in both groups was infection. Patients >75 had higher risk of NRM from infection, CRS, and other causes, but not from ICANS (16). Thus, these reports support the use of CAR T-cell therapies in older patients with primary refractory or early-relapsed disease and should be considered as a potentially curative treatment in this age group. It is also important to consider the fact that the median age at diagnosis for DLBCL is 67 years based on SEER data; thus, it is practical to understand the most effective and potentially less toxic treatment approaches for older DLBCL patients (3).

More recently, Lunning et al. performed a prospective observational study of older patients with R/R DLBCL who received >2 lines of therapy with ECOG performance status of 2 and were then treated with axi-cel (17); outcomes were compared to a matched chemoimmunotherapy control group from the database of the prior SCHOLAR-1 study, which originally reported poor outcomes for refractory DLBCL to next-line of therapy with ORR and mOS of 26% and 6.3 months, respectively (2). In Lunning et al.’s study group, 39% of patients were >65 years and 20% were >70 years. In their subgroup analysis, they reported longer OS in patients >65 years treated with axi-cel compared to chemoimmunotherapy (HR 0.32). They reported an ORR of 82% vs 25% and a CR of 68% vs 13% in patients >65 years treated with CAR T-cells vs chemoimmunotherapy, respectively. Similarly, Berning et al. reported a retrospective analysis of older patients with R/R DLBCL treated with either axi-cel or tisa-cel (18). They grouped patients as either <70 or >70 years at time of CAR T infusion. They reported similar ORRs between the two group (77.7% vs 78.3%, respectively) as well as similar mPFS (10.2 mo vs 11.1 mo). Notably, they found the mOS for <70 and >70 to be 21.8 months vs 34.4 months, although this difference was not statistically significant. Rates of grade >3 CRS and ICANs were similar among the groups as well (11.9% vs 7.9%, p=0.45, and 18.4% vs 22.2%, p=0.57, respectively). These data again support prior findings that older patients with DLBCL may derive greater benefit from CAR T-cell therapy compared to standard chemoimmunotherapy in the relapsed and refractory setting.

As we increase our knowledge and optimize management of CAR T-cell therapies, effective treatment option is becoming more accessible to a larger population of patients, particularly in older patients with limited functional status (i.e. ECOG 2) and comorbidities where autoHCT may not be an option. More recent observational and retrospective studies demonstrate safety and efficacy outcomes of CAR T-cell therapies in the older adult population (**Table 1**). Furthermore, the 5-year follow-up of the ZUMA-1 trial reported a 5-year OS of 42.7% in which 24% of the patients were >65, suggesting the curative potential of axi-cel in R/R large B-cell lymphomas (19). Therefore, it appears that for older patients, particularly those aged 65 to 74 with R/R DLBCL, CAR T-cell therapy should be strongly considered as a potentially curative treatment option. The improved outcomes in older patients is a surprising finding; however this data is primarily from subgroup analyses and retrospective studies, and not designed to directly compare subgroup responses stratified by age. It is possible that these outcomes in part due to selection bias, with younger patients in these cohorts representing particularly resistant disease whereas the older cohort reflects more of a ‘real world’ post-CAR T-cell outcomes (20).

**Table 1:** Efficacy and safety data for CAR T-cells in older adults.

Study/Author	Comparison/Intervention	Response	Survival	Adverse Events	Other	Ref #
ZUMA-1	Axi-cel in R/R DLBCL	ORR: ≥65 – 92% <65 – 79% CR: ≥65 – 75% <65 – 53%	mPFS ≥65 – 13.2 mo <65 – 5.6 mo	Grade >3 CRS: 13% Grade >3 ICANS: 31%		(6)
JULIET	Tisa-cel in R/R DLBCL	ORR: ≥65 – 59% <65 – 49%		Grade >3 CRS: 22% Grade >3 ICANS: 12%		(7)
TRANSCEND NHL-001	Liso-cel in R/R DLBCL	ORR: ≥65 – 75.9% <65 – 70.3% CR: ≥65 – 60.2% <65 – 48.0%		Grade >3 CRS: 1% Grade >3 ICANS: 13%		(8)
PILOT	Liso-cel as second-line therapy	ORR: <65 v >65 (66.7% v 81.8%) <70 v >70 (69.2% v 83.3%) <75 v >75 (75.8% v 85.7%) CR: <65 v >65 (50.0% v 54.4%) <70 v >70 (46.2% v 56.3%) <75 v >75 (57.6% v 50.0%)		Grade >3 CRS: <70 v >70 (0.0% v 2.0%) <75 v >75 (0.0% v 4.0%) Grade >3 ICANS: <70 v >70 (0.0% v 6.0%) <75 v >75 (6.0% v 4.0%)		(10)
ZUMA-7	Axi-cel vs SOC for second-line for patients >65	ORR CAR T – 88% SOC – 52% CR CAR T – 75% SOC – 33%		Grade >3 AEs CAR T – 94% SOC – 82%	Day 100 and Day 150 QOL scores higher in CAR T arm.	(11)
Akhtar et al.	CAR T vs AutoHCT for patients >65 (Retrospective)		1-year PFS: CAR T – 51% AutoHCT – 52% 1-year OS: CAR T – 73% AutoHCT – 71%			(15)
Guffroy et al.	CAR T in patients >75 (Restropective)	ORR: ≥75 – 74.8% <75 – 75.0% CR: ≥75 – 62.6% <75 – 60.8%	mPFS ≥75 – 8.2 mo <70 – 6.1 mo mOS ≥75 – 18.3 mo <70 – 24.0 mo	Grade >3 CRS: ≥75 – 7.3% <75 – 7.4% Grade >3 ICANS: ≥75 – 9.8% <75 – 12.4%	NRM: ≥75 – 19.5% <75 – 8.1% Early NRM (<28 days) ≥75 – 2.4% <75 – 1.2% Late NRM (≥28 days) ≥75 – 17.1% <75 – 6.9%	(16)
Lunning et al.	Axi-cel vs historic control of SOC for patients >65 treated with >2 lines of therapy (Prospective)	ORR: CAR T – 82% SOC – 25% CR: CAR T – 68% SOC – 13%				(17)
Berning et al.	Axi-cel or Tisa-cel (Retrospective)	ORR: ≥70 – 78.3% <70 – 77.7%	mPFS: ≥70 – 11.1 mo <70 – 10.2 mo mOS: ≥70 – 34.4 mo <70 – 21.8 mo	Grade >3 CRS: ≥70 – 7.9% <70 – 11.9% Grade >3 ICANS: ≥70 – 22.2% <70 – 18.4%		(18)

Axi-cel, Axicabtagene ciloleucel; DLBCL, Diffuse Large B-Cell Lymphoma; R/R, Relapsed/Refractory; ORR, Objective Response Rate; CR, Complete Response; mPFS, median Progression-Free Survival; CRS, Cytokine Release Syndrome; ICANS, Immune Effector Cell-Associated Neurotoxicity Syndrome; Tisa-cel, Tisagenlecleucel; Liso-cel, Lisocaptagene maraleucel; SOC, Standard of Care (chemoimmunotherapy); CAR T, Chimeric Antigen T-Cell Receptor T-cell Therapy; AEs, Adverse Events; QOL, Quality of Life; AutoHCT, Autologous Hematopoietic Stem Cell Transplantation; NRM, Non-Relapse Mortality.

## Bispecific antibodies

Bispecific Antibodies targeting both CD20 and CD3 have recently become established treatment options for R/R DLBCL. These include Glofitamab, Epcoritamab, Mosunetuzumab and Odronextamab. Data thus far have shown promising and durable response rates with low rates of CRS and ICANS, making bispecific antibody therapy a safe and effective option for elderly and unfit patients. Currently, both Glofitamab and Epcoritamab have FDA-approval for R/R DLBCL after at least two lines of therapy, including patients who previously received hematopoietic stem cell transplantation or CAR T-cell therapy.

Glofitamab was approved based on the phase II study by Dickinson, et al. (21) reporting on 155 patients with R/R DLBCL who had received at least two prior lines of therapy and were treated with glofitamab monotherapy. Subgroup analysis reported no difference in CR rate between patients <65 vs ≥ 65 (41% vs 38%). Rates of Grade 3 and 4 CRS were 3% and 1%, respectively. Grade ≥ 3 ICANS occurred in 3% of patients. Thus, glofitamab has shown similar efficacy in older patients ≥ 65 as compared to younger < 65, with low rates of CRS and ICANS. Recently, the STARGLO trial was presented at EHA2024 by Abramson et al. (22). This was a phase III randomized trial comparing the combination glofitamab with gemcitabine and oxaliplatin (G-GemOx) to rituximab-gemox (R-GemOx) in patients with R/R DLBCL who were not eligible for autoHCT. They enrolled 183 to the G-GemOx arm and 91 patients to the R-GemOx arm with a median age of 68 and >60% of patients being >65 years. They reported improved primary endpoints of mOS and mPFS for G-GemOx compared to R-GemOx (mOS 25.5 months vs. 12.9 months and mPFS 13.8 months vs 3.6 months). G-GemOx also showed improved ORR and CR at 68.3% and 58.5% compared to R-GemOx at 40.7% and 25.3%, respectively. Subgroup analysis showed similar benefits with G-GemOx regardless of age group (<65 vs >65). Rates of grade >3 AEs were higher in the G-GemOx group compared to the R-GemOx group, at 77.8% vs 40.9%. The rate of grade >3 CRS in the G-GemOx group was 2.3%, primarily in the first cycle. Thus, glofitamab is an effective and safe treatment option for patients >65 with R/R disease and may possibly be more effective in combination with chemotherapy.

The EPCORE NHL-1 trial was a Phase I/II dose escalation/expansion trial that lead to the approval of epcoritamab in treating R/R aggressive B-cell lymphomas. They reported on 157 patients, of whom 88.5% had DLBCL and a median age of 64. Subgroup analysis reported ORR for patients <65, 65-74, and >75 of 56.3%, 68.8% and 72.4%, respectively. CR rates were 35.0%, 39.6%, and 48.3%, respectively. Grade ≥ 3 CRS and ICANS occurred at 2.5% and 0.6%, respectively (23). Though not statically significant, it appears that elderly patients >65 years may receive the greatest benefit from epcoritamab in the R/R setting with manageable rates of CRS and ICANS.

Though not currently FDA approved for DLBCL (approved for R/R follicular lymphoma), mosunetuzumab has specifically been studied in elderly patients by Olszewski, et al. They evaluated the safety and efficacy of mosunetuzumab in the front-line treatment for elderly or unfit patients for SOC chemoimmunotherapy. They enrolled patients into two age groups, 60-79 years and ≥ 80 years, who had other comorbidities or limitations that prevented their candidacy for standard chemoimmunotherapy. They recently reported an updated 1-year follow-up of their Phase I/II study in which the median age of the patients was 83 years and after a median of 8 cycles and median follow-up of 23.3 months, the ORR and CR rates were 56% and 43%, respectively. The median duration of CR was 15.8 months. There was no grade >3 CRS and no reports of ICANS (24). More recently, mosunetuzumab is being studied in combination with the antibody-drug conjugate, polatuzumab vedotin, by Olszewski, et al. as front-line treatment for elderly/unfit patients with DLBCL. They again enrolled patients into two age groups, 60-79 years and ≥ 80 years. With median age of 81 years, preliminary results for ORR and CR have been reported at 55% and 45%, respectively. CRS was common, and all events resolved within a median of 2 days (25). Current guidelines recommend lower intensity chemoimmunotherapy for elderly and unfit patients, such as R-mini-CHOP, in which retrospective studies have reported ORR and CR rates of 79% and 53%, respectively (26). Thus, with ORRs and CRs ranging from 55-56% and 43-45%, respectively, for upfront mosunetuzumab both as monotherapy and in combination with antibody-drug conjugates, further investigation is needed to fully evaluate the benefits of bispecific antibody use in the front-line setting.

In the R/R setting, mosunetuzumab was evaluated by Bartlett, et al. in their Phase I/II study. With a median age of 66.5 years, they reported an ORR and CR of 42.0% and 23.9%, respectively. There were no age-related sub-group analyses reported in their study (27). Similarly, mosunetuzumab is being evaluated in combination with polatuzumab vedotin in R/R large B-cell lymphomas by Budde et al. Preliminary reports show that, with a median age of 68.0 years, 87.8% of patients with DLBCL have ORR and CR rates of 62.2% and 50%, respectively. The rate of any grade CRS was 18.4% (28).

Lastly, the ELM-1 study evaluated odronextamab in R/R NHL. This is a phase I dose-escalation study in which the median age was 67 years with 59% of patients being ≥ 65. Of the 145 patients enrolled, 85 patients had a diagnosis of DLBCL (58.6%). Though there was no age-related subgroup analysis, subgroup analysis was reported based on prior CAR T-cell therapy. For those patients with DLBCL who did not receive prior CAR T-cell therapy, ORR was 53%, with all responses being CR for those patients who received doses of 80 mg or higher. For those patients who had received prior CAR T-cells, ORR was 33% with CR of 27%. Overall rates of grade ≥ 3 CRS and ICANS were 7% and 3%, respectively (29). Thus, odronextamab seems to have similar response rates and rates of CRS/ICANS as other bispecific antibody therapies, but possibly higher CR rates, especially especially in those who have not received prior CAR T therapy. Further subgroup analysis is needed to assess specific efficacy in elderly and unfit patients.

The use of single agent bispecific antibody therapies provides an effective and safe option for elderly/unfit patients, especially in the R/R setting. With lower rates of CRS/ICANS, the side effect profiles become much safer and tolerable in comparison to CAR T-cell therapy for this specific patient population. Both glofitamab and epcoritamab have received FDA-approval in the third-line setting. Thus far, there appears to be substantial benefit be in the R/R setting, even in those that have failed CAR T-cell therapy. Current studies are further assessing safety and efficacy of bispecifics in combinations with antibody-drug conjugates, as well as in combination with cellular therapies (i.e. preceding CAR T-cell therapy) and chemotherapy. **Table 2** outlines the available evidence supporting the safety and efficacy of bispecific antibody therapy. Further studies and subgroup analyses are needed to better understand and optimize the benefits of bispecific antibody therapies for elderly DLBCL patients, but they may be a more accessible and less toxic option.

**Table 2:** Safety and efficacy of bispecific antibody therapies.

Study/Author	Comparison/Intervention	Response	Survival	Adverse Events	Other	Ref #
Dickinson et al.	Glofitamab	CR ≥65 – 38% <65 – 41%		Grade 3 CRS: Overall – 3% Grade 4 CRS: Overall – 1% Grade ≥ 3 ICANS: Overall – 3%		(21)
STARGLO	Glofitamab + GemOx vs R-GemOx in patients unfit for AutoHCT	ORR Glofit-GemOx -68.3% R-GemOx -40.7% CR Glofit-GemOx -58.5% R-GemOx -25.3%	mOS Glofit-GemOx -25.5 mo R-GemOx -12.9 mo mPFS Glofit-GemOx -13.8 mo R-GemOx -3.6 mo	Grade >3 AEs: Glofit-GemOx: 77.8% R-GemOx: 40.9% Grade >3 CRS Glofit-GemOx: 2.3%		(22)
EPCORE NHL-1	Epcoritamab for R/R disease	ORR <65 – 56.3% 65-75 – 68.8% >75 – 72.4% CR <65 – 35.0% 65-75 – 39.6% >75 – 48.3%		Grade >3 CRS: Overall – 2.5% Grade >3 ICANS: Overall – 0.6%		(23)
Olszewski et al.	Mosunetuzumab for front-line in elderly 60-80yrs	ORR – 56% CR – 43% mDO(CR) – 15.8 mo		No grade >3 CRS No grade >3 ICANS		(24)
Olszewski et al.	Mosunetuzumab + Polatuzumab vedotin for front-line in elderly/unfit	Preliminary: ORR – 55% CR – 45%				(25)
Bartlett et al.	Mosunetuzumab for R/R disease	Overall: ORR – 42.0% CR – 23.9% (no age-related subgroup analysis)				(27)
Budde et al.	Moasunetuzumab + Polatuzumab vedotin for R/R disease	Preliminary: ORR – 62.2% CR – 50%		Any grade CRS – 18.4%		(28)
ELM-1	Odronextamab for R/R NHL	No prior CAR T ORR - 53% (all CR) Prior CAR T ORR – 33% CR – 27%		Grade >3 CRS: Overall – 7.0% Grade >3 ICANS: Overall – 3.0%		(29)

CR, Complete Response; CRS, Cytokine Release Syndrome; ICANS, Immune Effector Cell-Associated Neurotoxicity Syndrome; R, Rituximab; GemOx, Gemcitabine + Oxaliplatin; AutoHCT, Autologous Hematopoietic Stem Cell Transplantation; ORR, Objective Response Rate; mOS, median Overall Survival; mPFS, median Progression-Free Survival; AEs, Adverse Events; R/R, Relapsed/Refractory; mDO(CR), median Duration of Complete Response; NHL, Non-Hodgkin Lymphoma; CAR T, Chimeric Antigen T-Cell Receptor Therapy.

## High-dose chemotherapy and autologous stem cell transplantation

Prior to the advent of CAR T-cell therapy, high-dose chemotherapy and autoHCT was the SOC curative-intent treatment for patients with R/R DLBCL. The PARMA study established PFS and OS benefit of high-dose chemotherapy and autoHCT as compared to conventional chemotherapy in patients with relasped chemo-sensistive NHL (30). The five-year OS was 53% in the transplantation group compared to 32% in the conventional treatment group; EFS was 46% with autoHCT versus 12% with conventional treatment. Rates of toxicity and infection were higher in the autoHCT group, and no patients in the conventional therapy group died from toxicity-related events. The superior EFS and OS with autoHCT compared to conventional therapy established this treatment as the SOC for eligible patienets with R/R DLBCL. The median age in the PARMA trial was 43, with the upper age limit being 60, providing limited data on outcomes in older patients. In high-risk DLBCL patients in the pre-rituximab era, autoHCT was also considered as consolidation in first remission; however, this approach did not show benefit when compared to rituximab in addition to chemotherapy (31–33).

The potential for cardiovascular, gastrointestinal, infectious, and other toxicities due to conditioning chemotherapy are a primary concern in older patients with R/R DLBCL being considered for high-dose chemotherapy and autoHCT. Furthermore, non-relapse mortality (NRM) or treatment-related mortality (TRM) is a major factor in the decision to proceed with autoHCT. Several variables that may affect NRM/TRM and toxicity have been examined in retrospective analyses, including risk stratification categories such as the hematopoietic cell transplantion-specific comorbidity index (HCT-CI), response to salvage therapy, disease status at the time of transplantation, and age 60 or older at the time of autoHCT.

Several retrospective studies have analyzed TRM in older patients undergoing high-dose chemotherapy and autoHCT for NHL (**Table 3**). Specifically in the DLBCL population, Jantunen et al. utilized the European Blood and Marrow Transplantation Registry to analyze outcomes in a cohort of 2612 patients with R/R DLBCL from 2000 to 2005; 18% of these patients were >60 years (34). They found that the older population received at least two prior lines of therapy, were less commonly in first remission at the time of transplantation, and also underwent autoHCT later after their diagnosis, 14 months versus 7.5 months for patients <60 years. NRM was higher in patients >60 versus <60; 4.4% vs 2.8% at 100 days, 8.7% vs 4.7% at 1year, and 10.8% vs 6.5% at 3 years. Three-year PFS and OS were also lower in the older vs younger population, 51% vs 62%, and 60% vs 70%, respectively. Munshi et al. reported outcomes for autoHCT patients aged 60-69 versus >70 with R/R DLBCL undergoing BEAM, with or without rituximab, using data from the Center for International Blood and Marrow Transplant Research data from 2008 to 2019 (35). On univariate analysis, 100-day NRM was not significantly different in the two cohorts, 3% vs 4%, respectively, in the 60-69 and >70 groups. The 1-year NRM was also similar, 6% vs 8%, respectively. Incidence of relapse/progression at 5 years was not statistically significant between the 60-69 and >70 cohorts, at 47% and 45%, respectively. Multivariate analysis also showed no significant difference in NRM or relapse in patients aged 60-69 vs >70 years. PFS was also not significantly different between the two groups. There was, however, a statistically significant difference in OS at five years, 55% in the 60-69 cohort versus 41% in the >70 cohort (p=0.02). With additional analysis of infections and cause of death, this was attributed mainly to inferior post-relapse survival in the >70 group. A small single-center study by Bitran et al. reported on 11 patients undergoing high-dose chemotherapy and autoHCT between 1995 and 2002 (36). The 1-year TRM was 9% and the median disease-free survival was 17 months. This was in comparison to a 1-year TRM of 2.5% in a cohort of 78 patients under age 65 receiving the same treatment during this time period. The 1-year DFS for patients >65 and <65 was 67% vs 74%, respectively, and OS was 67% and 81%, respectively. Although TRM was greater in patients >65, DFS and OS were similar.

**Table 3:** Retrospective analyses of older adults with NHL treated with high-dose chemotherapy and autoHCT.

Author	Year of Publication	Age and Number of patients	NHL Subtypes and % DLBCL	Conditioning Regimen(s)	PFS	OS	100-Day TRM/NRM	Late TRM/NRM	Reference Number
Jantunen et al.	2008	≥60y – 416 <60y - 2149	DLBCL – 100%	NR	3-yr PFS: ≥60 – 51% <60y – 62%	3-yr OS: ≥60y – 60% <60y – 70%	100-day NRM: ≥60y – 4.4% <60y – 2.8%	1-yr NRM: ≥60y – 8.7% <60y – 4.7% 3-yr NRM: ≥60y – 10.8% <60y – 6.5%	(34)
Munshi et al.	2022	60-69y – 363 ≥70y - 103	DLBCL – 100%	BEAM	5-yr PFS: 60-69 – 40% ≥70 – 38%	5-yr OS: 60-69 – 55% ≥70 – 41%	NR	NR	(35)
Bitran et al.	2003	≥65y - 11	Large B-cell Lymphoma	TBI-CyEtop – 55% BEAM – 45%	4-yr DFS – 44%	4-yr OS – 44%	100-day TRM – 9%	NR	(36)
Dahi et al.	2014	≥60y – 202	DLBCL – 37% MCL – 34% FL – 6% CNSL – 7% TCL – 12% Other – 4%	BEAM – 74% R-BEAM – 5% RIT-BEAM – 6% Other – 15%	3-yr PFS: 60%	3-yr OS: 73%	4%	4%	(37)
Hermet et al.	2015	≥70y - 81	DLBCL – 50% FL – 20% MCL – 19% TCL – 6% Other – 5%	BEAM – 75% Melphalan – 17%	mPFS – 21mo	mOS – 43 mo	100-day NRM: 5.4%	1-yr NRM: 8.5%	(38)
Lemieux et al.	2019	≥60y - 90	DLBCL – 38% FL – 36% MCL – 20%	BEAM – 44% BEAC – 50% BuCyEtop – 6%	5-yr PFS: 47% mPFS: 56mo	5-yr OS: 60% mOS: not reached	100-day NRM: 1%	1-yr NRM: 1%	(39)
Martin et al.	2015	≥65y - 73	DLBCL	BEAM – 100%	2-yr PFS: 67.2% mPFS: not reached	2-yr OS: 78.5% mOS: 90 mo	100-day TRM: 2.7%		(40)
Sun L et al.	2018	≥70y – 107	DLBCL – 58% FL – 7% TCL – 17% MCL – 10% HL – 5% PCNSL – 3%	CBV – 44% BEAM – 31% BuCy – 24% Other – 1%	2-yr PFS: 58%	2-yr OS: 65%	100-day TRM: 2%	1-yr NRM: 5% 2-yr NRM: 7%	(41)
Gohil et al.	2015	≥60y – 81	DLBCL – 25% MCL – 23% FL – 17% HL – 11% TCL – 10% Other – 14%	BEAM or LEAM	5-yr PFS: Overall - 49% ≥65 – 27% 60-64 – 57%	5-yr OS: Overall – 54% ≥65 – 47% 60-64 – 57%	100-day NRM: 1.3%	1-yr NRM: 2.5% 2-yr NRM: 5.1% 5-yr NRM: 10.8%	(42)
Jantunen et al.	2006	>60y - 88	DLBCL – 33% MCL – 31% FL – 17% TCL – 14% Other – 5%	BEAC – 56% BEAM – 39% TBI-Cy – 5%	2-yr PFS: Overall – 62% 5-yr PFS: Overall – 45%	2-yr OS: Overall – 63% 5-yr OS: Overall – 44% DLBCL – 38%	100-day TRM: Overall - 11% BEAM – 18% BEAC – 9% DLBCL – 10%	>100-day TRM – 7% 4-yr NRM – 19%	(43)
Gopal et al.	2001	≥60y - 53	Aggressive – 83% Chemosensitive – 75%	BuMelTh – 45% TBI-CyEtop – 30% TBI-Cy – 15% Other – 10%	4-yr PFS – 24%	4-yr OS: Overall – 33% Chemosens – 39% Chemoresist – 15%	100-day TRM – 9.4%	1-yr TRM – 17.4% 3-yr TRM – 22.4%	(44)
Wildes et al.	2008	≥60y – 59 <60y - 93	NHL	BEAM – 100%	mDFS: ≥60y – 21.8 mo <60y – 29.9 mo	mOS: ≥60y – 47.7 mo <60y – 62.5 mo	100-day TRM: ≥60y – 8.5% <60y – 4.5%	NR	(45)
Elstrom et al.	2011	≥69y - 21	DLBCL – 62%	BCV or BEAM	mPFS – 8 mo	mOS – 18 mo	100-day TRM: 19%	NR	(46)
Andorsky et al.	2011	≥70y – 17 65-69y - 39	NHL	NR	NR	OS decreased in >70 (HR 1.98) mOS: ≥70y – 2.6 yr 65-69y – 6 yr	100-day NRM: Increased in >70 (HR 6.04) ≥70y – 17.65% 65-69y – 5.13%	1-yr NRM: ≥70y – 35% 65-69y – 8%	(47)
Hosing at al.	2008	≥65y - 99	DLBCL – 53% MCL – 15% FL – 11% TCL – 4% Other – 17%	BEAM – 42% R-BEAM – 35% Cy/TBI – 10% RIT-BEAM – 2%	NR	3-yr OS: 61%	26-mo NRM: 8%	36-mo NRM: 12%	(48)
Wudhiukarn et al.	2023	≥65y 492 65-70y 192 ≥70y 130	de Novo DLBCL – 41% Transformed DLBCL – 18.3% Primary CNS lymphoma – 7.5% MCL – 13.2% Indolent B -cell NHL – 0.4% T-cell NHL – 12.2% HL 0.6%	BEAM – 90% Thiotepa-Carmustine – 9.1% High-dose melphalan – 0.8%	3-year PFS 52.2% 5-year PFS 43.9%	Overall 3-year OS 63.9% Overall 5-year OS 54.1% 3-year OS 65-70 73.6% 3-year OS >70 67.4% 5-year OS 65-70 56.9% 5-year OS >70 49.7%	Overall 100-day NRM: 4.1% 65-70: 3.7% >70: 4.6%	Overall 1-year NRM: 6% 65-70: 4.7% >70: 7.8% Overall 3-year NRM 10.2% 65-70: 9.1% >70: 12%	(49)

NR, Not Reached.

Several other retrospective studies have analyzed outcomes of autoHCT in older patients with NHL, inclusive of but not limited to DLBCL. The 100-day TRM or NRM varies widely from 1% to 19% (37–49). As demonstrated by the range of reported 100-day TRM, some of these studies found the impact of age on TRM to be non-significant, while others found increased risk of toxicities and TRM in older patients. Hosing et al. reported on 99 patients with relapsed NHL (53% DLBCL) aged 65 or older and found that HCT-CI did not impact overall survival but was associated with increased risk of grade 3-5 toxicities (48). Contrastingly, Elstrom et al. found that high-risk HCTI-CI score was associated with inferior OS and higher TRM (46).

Disease status at the time of autoHCT has also been evaluated in relation to survival. Lemieux et al. reported worse PFS and OS in patients with progressive disease (PD) or stable disease (SD) versus those with complete or partial response at time of transplanatation, with HR 5.0 for PFS and HR 6.52 for OS (39). Studies by Gopal et al. Hosing et al. also reported that PD or SD at the time of autoHCT is associated with inferior OS in older patients with NHL (44, 48).

Several of these studies further categorized patients into subgroups by age. Results from Munshi et al. are reported above (35), and Dahi et al. similarly reported PFS and OS outcomes in patients 60-64 and >65 without any significant differences (37). Lemieux et al. found no difference in 5-year PFS and OS or in time to engraftment in patients 60-64 versus >65 years (39). Patients aged 60-64 were more likely to receive BEAM preparative regimen (versus BEAC or Bus/Cy/Etoposide) and to receive total parenteral nutrition and narcotic medications compared to the >65 subgroup, possibly highlighting the toxicity related to BEAM conditioning. Gohil et al. analyzed patients aged 60 and older and subcategorized them into those aged 60-64 and 65 and older (42). The >65 group had significantly worse 5-year PFS and OS compared to those aged 60-64, 27.6% vs 57.7%, and 47.6% vs 57.7%, respectively. This difference was contributed mainly to increased relapse risk in the >65 group, 53.8%, vs 30.1% in the 60-64 group. 100-day and 1-year NRM were not significantly different, 1.8% vs 0% and 3.6% vs 0%, respectively. Wudhikarn et al. reported outcomes of AutoHCT with R/R NHL in patients aged 65-70 and >70 between 2000 and 2021 (49). The overall 100-day NRM was 4.1% for the entire cohort, and 3.7% in patients aged 65-70 vs 4.6% in those >70 years. In subgroup analysis, NRM in the >70 cohort was lower in more recent years: 6.8% from 2000 to 2008 vs 3.6% from 2009 to 2021. Both the 3-year and 5-year OS were superior in the younger cohort compared to the older cohort: 73.6% vs 67.4% and 56.9% vs 49.7%, respectively. Heterogeneity in outcomes is again demonstrated here in further subgroups of older patients undergoing autoHCT for R/R NHL including DLBCL.

## Second primary malignancies

The possibility of second primary malignancies (SPM) is also a consideration when counseling patients on the potential risks associated with cellular therapies including high-dose chemotherapy and autoHCT or CAR T-cell therapies. Dahi et al. found that SPM, consisting of either myelodysplastic syndrome or acute myeloid leukemia, occurred in 4% of patients aged 60 or older with NHL after autoHCT, with a rate of 10% in those aged 70 and older versus 2% in those ages 60-69 (37). In Wudhikarn’s Mayo cohort analysis, SPM was the second most common cause of death after relapse, accounting for 13.5% of all deaths. Of the 28 total SPM, 20 were myeloid neoplasms (49).

More recently, the risk of SPM has been described with longer term follow up of the current FDA-approved CAR T-cell therapies. Hamilton et al. reported 26 SPM in a total of 791 CAR T-cell infusions. Fourteen of these were hematologic malignancies, including 13 cases of either MDS or AML and 1 case of a T-cell lymphoma (50). The cumulative incidence of second primary hematologic tumors at 3-year follow up after CAR T-cell infusions was 6.5%. Ghilardi et al. also reported on 449 patients treated with CD19- and BCMA-directed CAR T-cell therapies at the University of Pennsylvania (51). They identified a total of 16 (3.6%) SPM at a median follow up of 10.3 months. Only one case of a T-cell lymphoma was identified. Further investigation into the incidence of SPM, in particular T-cell lymphomas and the risk of viral vector integration, is ongoing with longer term follow up of CAR T-cell therapies. Patients should be counseled on the risk of SPM following these cellular therapies.

## Discussion

Cellular therapies have transformed the landscape of treatment for R/R DLBCL, a disease that historically had poor outcomes with chemotherapy alone. Older adults have limited treatment options due to their ability to tolerate traditional treatments for R/R DLBCL, such as salvage chemotherapy and high-dose chemotherapy with autoHCT. With the approval of numerous CAR T-cell products and bispecific antibodies, as well as improvements in the tolerability of autoHCT in older patients, these treatments should be considered carefully in the older adult population. The overall fitness and functional status of the patient, their comorbidities, disease risk stratification, disease status, and access to a transplant centers should all be evaluated when considering cellular therapies for an older adult with R/R DLBCL. Age alone should not be a limiting factor for these potentially curative treatments.

## References

[1] Sehn L Salles G. Diffuse large B-cell lymphoma. N Engl J Med. (2021) 384:842–58. doi: 10.1056/NEJMra2027612 PMC8377611 33657296

[2] Crump Neelapu SS Farooq U Van Den Nest E Kuruvilla A Westin J. Outcomes in refractory diffuse large B-cell lymphoma: results from the international SCHOLAR-1 study. Blood. (2017) 139:1800–8. doi: 10.1182/blood-2017-03-769620 PMC5649550 28774879

[3] Available online at: https://seer.cancer.gov/statfacts/html/dlbcl.html (accessed July 01, 2024).

[4] Lewis JH Kilgore ML Goldman DP Trimble EL Kaplan R Montell MJ. Participation of patients 65 years of age or older in cancer clinical trials. J Clin Oncol. (2003) 21. doi: 10.1200/JCO.2003.08.010 12663731

[5] Bumanlag IM Jaoude JA Rooney MK Taniguchi CM Ludmir EB. Exclusion of older adults from cancer clinical trials: review of the literature and future recommendations. Semin Radiat Oncol. (2022) 32:125–34. doi: 10.1016/j.semradonc.2021.11.003 PMC8944215 35307114

[6] Neelapu SS Locke FL Bartlett NL Lekakis LJ Miklos DB Jacobson CA. Axicabtagene ciloleucel CAR T-cell therapy in refractory large B-cell lymphoma. N Engl J Med. (2017) 377:2531–44. doi: 10.1056/NEJMoa1707447 PMC5882485 29226797

[7] Schuster SJ Bishop MR Tam CS Waller EK Borchmann P McGuirk JP. Tisagenlecleucel in adult relapsed or refractory diffuse large B-cell lymphoma. N Engl J Med. (2019) 380:45–56. doi: 10.1056/NEJMoa1804980 30501490

[8] Abramson JS Palomba ML Gordon LI Lunning MA Wang M Arnason J. Lisocabtagene maraleucel for patients with relapsed or refractory large B-cell lymphomas (TRANSCEND NHL 001): a multicentre seamless design study. Lancet. (2020) 396:839–52. doi: 10.1016/S0140-6736(20)31366-0 32888407

[9] Thompson J Schneider B Brahmer J Armand P Zaid M Achufusi A. Management of Immunotherapy-Related Toxicities. NCCN Guidelines Version 1.2024. J Natl Compr Cancer Netw. (2023).

[10] Sehgal A Hoda D Riedell PA Ghosh N Hamadani M Hildebrandt GC. Lisocabtagene maraleucel as second-line therapy in adults with relapsed or refractory large B-cell lymphoma who were not intended for haematopoietic stem cell transplantation (PILOT): an open-label, phase 2 study. Lancet Oncol. (2022) 23:1066–77. doi: 10.1016/S1470-2045(22)00339-4 35839786

[11] Westin JR Locke FL Dickinson M Ghobadi A Elsawy M van Meerten T. Safety and efficacy of axicabtagene ciloleucel versus standard of care in patients 65 years of age or older with relapsed/refractory large B-cell lymphoma. Clin Cancer Res. (2023) 29:1894–905. doi: 10.1158/1078-0432.CCR-22-3136 PMC10183830 36999993

[12] Johnson PC Neckermann I Sadrzadeh H Newcomb R El-Jawahri AR Frigault MJ. Clinical outcomes and toxicity in older adults receiving chimeric antigen receptor T cell therapy. Transplant Cell Ther. (2024) 30:490–9. doi: 10.1016/j.jtct.2024.02.019 38412928

[13] Chihara D Liao L Tkacz J Franco A Lewing B Kilgore KM. Real-world experience of CAR T-cell therapy in older patients with relapsed/refractory diffuse large B-cell lymphoma. Blood. (2023) 142:1047–55. doi: 10.1182/blood.2023020197 37339585

[14] Shadman M Pasquini M Ahn KW Chen Y Turtle CJ Hematti P. Autologous transplant vs chimeric antigen receptor T-cell therapy for relapsed DLBCL in partial remission. Blood. (2022) 139:1330–9. doi: 10.1182/blood.2021013289 PMC8900276 34570879

[15] Akhtar OS Cao B Wang X Torka P Al-Jumayli M Locke FL. CAR T-cell therapy has comparable efficacy with autologous transplantation in older adults with DLBCL in partial response. Blood Adv. (2023) 7:5937–40. doi: 10.1182/bloodadvances.2023010127 PMC10562759 37236167

[16] Guffroy B Bachy E Di Blasi R Guedon H Le Bras F Yakoub-agha I. Real-world Assessment of Anti-CD19 CAR-T Cells in Patients Aged 75 Years And Older With Relapsed Or Refractory Large B Cell Lymphoma: A LYSA Study From The DESCAR-T Registry. Presented at EHA2024. 2024 June 13-16. Madrid: European Hematology Association.

[17] Lunning MA Wang HL Hu ZH Locke FL Siddiqi T Jacobson CA. Benefit of axicabtagene ciloleucel versus chemoimmunotherapy in older patients and/or patients with poor ECOG performance status with relapsed or refractory large B-cell lymphoma after 2 or more lines of prior therapy. Am J Hematol. (2024) 99:880–9. doi: 10.1002/ajh.27283 PMC11665240 38504387

[18] Berning P Shumilov E Maulhardt M Boyadzhiev H Kerkhoff A Call S. Chimeric antigen receptor-T cell therapy shows similar efficacy and toxicity in patients with diffuse large B-cell lymphoma aged 70 and older compared to younger patients: A multicenter cohort study. Hemasphere. (2024) 8:e54. doi: 10.1002/hem3.54 38510993 PMC10953542

[19] Neelapu SS Jacobson CA Ghobadi A Miklos DB Lekakis LJ Oluwole OO. Five-year follow-up of ZUMA-1 supports the curative potential of axicabtagene ciloleucel in refractory large B-cell lymphoma. Blood. (2023) 141:2307–15. doi: 10.1182/blood.2022018893 PMC10646788 36821768

[20] Dreger P Holtick U Subklewe M von Tresckow B Ayuk F Wagner E. German Lymphoma Alliance (GLA); German Stem Cell Transplantation Registry (DRST). Impact of age on outcome of CAR-T cell therapies for large B-cell lymphoma: the GLA/DRST experience. Bone Marrow Transplant. (2023) 58:229–32. doi: 10.1038/s41409-022-01867-4 PMC9902271 36418916

[21] Dickinson MJ Carlo-Stella C Morschhauser F Bachy E Corradini P Iacoboni G. Glofitamab for relapsed or refractory diffuse large B-cell lymphoma. N Engl J Med. (2022) 387:2220–31. doi: 10.1056/NEJMoa2206913 36507690

[22] Abramson JS Ku M Hertzberg M Fox CP Herbaux C Vicente MF. Glofitamab plus Gemcitabin and Oxaliplatin (Glofit-GemOx) for Relapsed/Refractory (R/R) Diffuse Large B-cell Lymphoma (DLBCL): Results of a Global Randomized Phase III Trial (STARGLO). Presented at EHA2024. 2024 June 13-16. Madrid: European Hematology Association.

[23] Thieblemont C Phillips T Ghesquieres H Cheah CY Clausen MR Cunningham D. Epcoritamab, a novel, subcutaneous CD3xCD20 bispecific T-cell-engaging antibody, in relapsed or refractory large B-cell lymphoma: dose expansion in a phase I/II trial. J Clin Oncol. (2023) 41:2238–47. doi: 10.1200/JCO.22.01725 PMC10115554 36548927

[24] Olszewski A Avigdor A Babu S Levi I Eradat H Abadi U. Mosunetuzumab monotherapy continues to demonstrate promising efficacy and durable complete responses in elderly/unfit patients with previously untreated diffuse large B-cell lymphoma. Blood. (2022) 140:1778–80. doi: 10.1182/blood-2022-157768

[25] Olszewski A Eradat H Avigdor A Horowitz N Babu S Levi I. Mosunetuzumab and polatuzumab vedotin demonstrates preliminary efficacy in elderly unfit/frail patients with previously untreated diffuse large B-cell lymphoma. Blood. (2023) 142:855. doi: 10.1182/blood-2023-177588

[26] Guerra VA Ocampo M Cusnir M. Outcomes of R-mini-CHOP therapy for elderly patients with diffuse large B-cell lymphoma and high-grade follicular lymphoma. Blood. (2021) 138:4581. doi: 10.1182/blood-2021-153702

[27] Bartlett NL Assouline S Giri P Schuster SJ Cheah CY Matasar M. Mosunetuzumab monotherapy is active and tolerable in patients with relapsed/refractory diffuse large B-cell lymphoma. Blood Adv. (2023) 7:4926–35. doi: 10.1182/bloodadvances.2022009260 PMC10463194 37067952

[28] Budde L Olszewski A Assouline S Lossos I Diefenbach C Kamdar M. Mosunetuzumab plus polatuzumab vedotin demonstrates a favorable safety profile and efficacy in patients with relapsed or refractory large B-cell lymphoma: primary analysis of a phase ib/II study. Blood (2023) 142(1):613. doi: 10.1182/blood-2023-174209 37590026

[29] Bannerji R Arnason JE Advani RH Brown JR Allan JN Ansell SM. Odronextamab, a human CD20×CD3 bispecific antibody in patients with CD20-positive B-cell Malignancies (ELM-1): results from the relapsed or refractory non-Hodgkin lymphoma cohort in a single-arm, multicentre, phase 1 trial. Lancet Haematol. (2022) 9:e327–39. doi: 10.1016/S2352-3026(22)00072-2 PMC10681157 35366963

[30] Philip T Guglielmi C Hagenbak C Somers R Van Der Lelie H Bron D. Autologous bone marrow transplantation as compared with salvage chemotehrapy in relapses of chemotherapy-sensitive non-hodgkin’s lymphoma. N Engl J Med. (1995) 333:1540–5. doi: 10.1056/NEJM199512073332305 7477169

[31] Martelli M Gherlinzoni F De Renzo A Zinzani PL De Vivo A Cantonetti M. Early autologous stem-cell transplantation versus conventional chemotherapy as front-line therapy in high-risk, aggressive non-Hodgkin’s lymphoma: an Italian multicenter randomized trial. J Clin Oncol. (2003) 21:1255–62. doi: 10.1200/JCO.2003.01.117 12663712

[32] Milpied N Deconinck E Gaillard F Delwail V Foussard C Berthou C. Initial treatment of aggressive lymphoma with high-dose chemotherapy and autologous stem-cell support. N Engl J Med. (2004) 350:1287–95. doi: 10.1056/NEJMoa031770 15044639

[33] Vitolo U Chiappella A Angelucci E Rossi G Liberati AM Cabras MG. Dose-dense and high-dose chemotherapy plus rituximab with autologous stem cell transplantation for primary treatment of diffuse large B-cell lymphoma with a poor prognosis: a phase II multicenter study. Haematologica. (2009) 94:1250–8. doi: 10.3324/haematol.2009.007005 PMC2738717 19586937

[34] Jantunen E Canals C Rambaldi A Ossenkoppele G Allione B Blaise D. Autologous stem cell transplantation in elderly patients (≥60 years) with diffuse large B-cell lymphoma: an analysis based on data in the European Blood and Marrow Transplantation registry. Haematologica. (2008) 93:1837–42. doi: 10.3324/haematol.13273 18838474

[35] Munshi PN Chen Y Ahn KW Awan FT Cashen A Shouse G. Outcomes of autologous hematopoietic cell transplantation in older aptients with diffuse large B cell lymphoma. Transplant Cell Ther. (2022) 28:487e1–e7. doi: 10.1016/j.jtct.2022.05.029 PMC9375438 35609865

[36] Bitran JD Klein L Link D Kosirog-Glowacki J Stewart C Raack D. High-dose myeloablative therapy and autologous peripheral blood progenitor cell transplantation for elderly patients (greater than 65 years of age) with relapsed large cell lymphoma. Biol Blood Marrow Transplant. (2003) 9:383–8. doi: 10.1016/s1083-8791(03)00099-5 12813446

[37] Dahi PB Tamari R. Devlin SM Favorable outcomes in elderly patients undergoing high-dose therapy and autologous stem cell transplantation for non-Hodgkin lymphoma. Biol Blood Marrow Transplant. (2014) 20:2004–9. doi: 10.1016/j.bbmt.2014.08.019 PMC5480286 25175794

[38] Hermet E Cabrespine A Guieze R Garnier A Tempescul A Lenain P. Autologous hematopoietic stem cell transplantation in elderly patients (≥70 years) with non-Hodgkin’s lymphoma: A French Society of Bone Marrow Transplantation and Cellular Therapy retrospective study. J Geriatric Oncol. (2015) 6:346–52. doi: 10.1016/j.jgo.2015.04.005 26116168

[39] Lemieux C Ahmad I Bambace NM Bernard L Cohen S Delisle JS. Outcome of autologous hematopoietic stem cell transplant in older patients with B cell lymphoma when selected for fitness and chemosensitive disease. Leuk Res. (2019) 79:75–80. doi: 10.1016/j.leukres.2019.01.002 30654975

[40] Martin N Borchiellini D Coso D Gastaud L Boscagli A Saudes L. High-dose chemotherapy with carmustine, etoposide, cytarabine and melphalan followed by autologous stem cell transplant is an effective treatment for elderly patients with poor-prognosis lymphoma. Leuk Lymphoma. (2015) 56:2379–87. doi: 10.3109/10428194.2014.1001987 25563428

[41] Sun L Li S El-Jawahri A Armand P Dey BR Fisher DC. Autologous stem cell transplantation in elderly lymphoma patients in their 70s: outcomes and analysis. Oncologist. (2018) 23:624–30. doi: 10.1634/theoncologist.2017-0499 PMC5947447 29284757

[42] Gohil SH Ardeshna KM Lambert JM Pule MA Mohamedbhai S Virchis A. Autologous stem cell transplantation outcomes in elderly patients with B cell non-Hodgkin lymphoma. Br J Haem. (2015) 171:197–204. doi: 10.1111/bjh.13561 26119524

[43] Jantunen E Itälä M Juvonen E Leppa S Keskinen L Vasala K. Autologous stem cell transplantation in elderly (>60 years) patients with non-Hodgkin’s lymphoma: a nation-wide analysis. Bone Marrow Transplant. (2006) 37:367–72. doi: 10.1038/sj.bmt.1705266 16415893

[44] Gopal AK Gooley TA Golden JB Maloney DG Bensinger WI Petersdorf WH. Efficacy of high-dose therapy and autologous hematopoietic stem cell transplantation for non-Hodgkin’s lymphoma in adults 60 years of age and older. Bone Marrow Transplant. (2001) 27:593–9. doi: 10.1038/sj.bmt.1702833 11319588

[45] Wildes TM Augustin KM Sempek D Zhang QL Vij R Dipersio JF. Comorbidities, not age, impact outcomes in autologous stem cell transplant for relapsed non-Hodgkin lymphoma. Biol Blood Marrow Transplant. (2008) 14:840–6. doi: 10.1016/j.bbmt.2008.05.002 18541205

[46] Elstrom RL Martin P Rua SH Shore TB Furman RR Ruan J. Autologous stem cell transplant is feasible in very elderly patients with lymphoma and limited comorbidity. Am J Hematol. (2012) 87:433–5. doi: 10.1002/ajh.23108 22367772

[47] Andorsky D Cohen M Naeim A. Outcomes of auto-SCT for lymphoma in subjects aged 70 years and over. Bone Marrow Transplant. (2011) 46:1219–25. doi: 10.1038/bmt.2010.289 21151188

[48] Hosing C Saliba RM Okoroji GJ Popat U Couriel D Ali T. High-dose chemotherapy and autologous hematopoietic progenitor cell transplantation for non-Hodgkin’s lymphoma in patients >65 years of age. Ann Oncol. (2008) 19:1166–71. doi: 10.1093/annonc/mdm608 PMC4112363 18272911

[49] Wudhikarn K Johnson BM Inwards DJ Porrata LF Micallef IN Ansell SM. Outcomes of older adults with non-Hodgkin lymphoma undergoing autologou stem cell transplantation: a Mayo Clinic cohort analysis. TCT. (2023) 29. doi: 10.1016/j.jtct.2022.12.011 36563788

[50] Hamilton MP Sugio T Noordenbos T Shi S Bulterys PL Liu CL. Risk of second tumors and T-cell lymphoma after CAR T-cell therapy. N Engl J Med. (2024) 390:2047–60. doi: 10.1056/NEJMoa2401361 PMC11338600 38865660

[51] Ghilardi G Fraietta JA Gerson JN Van Deerlin VM Morrissette JJD Caponetti GC. T cell lymphoma and second primary Malignancy risk after commercial CAR T cell therapy. Nat Med. (2024) 30:984–9. doi: 10.1038/s41591-024-02826-w 38266761

